# Development and validation of a prognostic nomogram for colorectal cancer after radical resection based on individual patient data from three large-scale phase III trials

**DOI:** 10.18632/oncotarget.21845

**Published:** 2017-10-12

**Authors:** Michitaka Honda, Koji Oba, Takashi Akiyoshi, Hiromichi Maeda, Kosuke Kashiwabara, Mitsuro Kanda, Shuhei Mayanagi, Toru Aoyama, Chikuma Hamada, Sotaro Sadahiro, Yosuke Fukunaga, Masashi Ueno, Junichi Sakamoto, Shigetoyo Saji, Takaki Yoshikawa

**Affiliations:** ^1^ Department of Minimally Invasive Surgical and Medical Oncology, Fukushima Medical University, Fukushima, Japan; ^2^ Department of Biostatistics, Graduate School of Medicine, The University of Tokyo, Tokyo, Japan; ^3^ Department of Gastroenterological Surgery, Gastroenterological Center, Cancer Institute Hospital, Japanese Foundation for Cancer Research, Tokyo, Japan; ^4^ Cancer Treatment Center, Kochi Medical School Hospital, Kochi University, Nankoku, Japan; ^5^ Department of Gastroenterological Surgery (Surgery II), Nagoya University Graduate School of Medicine, Nagoya, Japan; ^6^ Department of Surgery, Keio University, Tokyo, Japan; ^7^ Department of Surgery, Yokohama City University, Yokohama, Japan; ^8^ Faculty of Engineering, Tokyo University of Science, Tokyo, Japan; ^9^ Department of Surgery, Tokai University, Isehara, Japan; ^10^ Tokai Central Hospital, Kakamigahara, Japan; ^11^ Japanese Foundation for Multidisciplinary Treatment of Cancer, Tokyo, Japan; ^12^ Department of Gastrointestinal Surgery, Kanagawa Cancer Center, Yokohama, Japan

**Keywords:** prognostic nomogram, prediction model, colorectal cancer, overall survival, disease free survival

## Abstract

**Background:**

Few prediction models have so far been developed and assessed for the prognosis of patients who undergo curative resection for colorectal cancer (CRC).

**Materials and Methods:**

We prepared a clinical dataset including 5,530 patients who participated in three major randomized controlled trials as a training dataset and 2,263 consecutive patients who were treated at a cancer-specialized hospital as a validation dataset. All subjects underwent radical resection for CRC which was histologically diagnosed to be adenocarcinoma. The main outcomes that were predicted were the overall survival (OS) and disease free survival (DFS). The identification of the variables in this nomogram was based on a Cox regression analysis and the model performance was evaluated by Harrell's c-index. The calibration plot and its slope were also studied. For the external validation assessment, risk group stratification was employed.

**Results:**

The multivariate Cox model identified variables; sex, age, pathological T and N factor, tumor location, size, lymphnode dissection, postoperative complications and adjuvant chemotherapy. The c-index was 0.72 (95% confidence interval [CI] 0.66-0.77) for the OS and 0.74 (95% CI 0.69-0.78) for the DFS. The proposed stratification in the risk groups demonstrated a significant distinction between the Kaplan–Meier curves for OS and DFS in the external validation dataset.

**Conclusions:**

We established a clinically reliable nomogram to predict the OS and DFS in patients with CRC using large scale and reliable independent patient data from phase III randomized controlled trials. The external validity was also confirmed on the practical dataset.

## INTRODUCTION

Surgical resection has been the pivotal treatment for patients with colorectal cancer (CRC), and recent advances in total mesorectal excision and multidisciplinary therapy have improved the oncological outcomes of these patients [1–3]. However, despite achieving a potentially curative resection and the administration of adjuvant treatment, the recurrence rate remains at 20% to 40% after curative resection in patients with stage II or III CRC. Because recent progress on multimodality treatment could improve the curability of such patients, conducting an intensive follow-up after curative resection for CRC has been reported to improve the survival rate [4]. However, the optimum follow-up duration for such individuals is still unclear. It is therefore important to identify the recurrence risk for each patient and to establish a reasonable follow-up schedule from the perspective of cost effectiveness. Therefore, a system for predicting the prognosis or recurrence patterns on an individual basis is required.

A nomogram is a clinically useful tool for predicting the prognosis of patients or other clinical events for individuals and it has been widely applied in the field of medical oncology [5]. Recently, some nomograms to predict the oncological outcomes of patients with resectable CRC have been developed [6–8]. The first nomogram reported by Weiser et. al is considered to be an epochal tool for clinicians, however, it is also associated with some limitations. For example, it did not include a sufficient sample size for model derivation, no validation study was performed and it targeted only the relapse free survival of patients with colon cancer in at a single cancer specialized hospital. Although the recent most reliable tool developed by Valentini et al. showed good c-index using largest dataset from five clinical trials, this prediction model only focused on western patients with rectal cancer who underwent adjuvant either chemoradiotherapy or radiotherapy.

Our primary goal was to develop and validate nomograms for predicting the overall survival and recurrence in patients who underwent curative resection for CRC. The prognostic nomogram established in this study is the first one every established for an Asian population and it is also based on the largest-scale data ever utilized in comparison to those described in previous studies. This new prediction model should also greatly benefit surgeons in their clinical practice.

## RESULTS

The descriptive statistics of the derivation cohort (*n* = 5,530) and validation cohort (*n* = 2,263) are listed in Table 1. The proportion of pathological stage II and III disease were higher in the derivation cohort than in the validation cohort because the dataset for derivation contained individual data from specific clinical trials. The median follow-up was 5.0 years in the derivation cohort and 5.2 years in the validation cohort.

**Table 1:** Patients characteristics and number of events of derivation and validation cohort

	Derivation cohort(*N* = 5530)	Validation cohort(*N* = 2263)
Age (median [range])	60 [20–75]	64 [23–91]
Sex		
Male (%)	3104 (56.1)	1252 (55.3)
Female (%)	2426 (43.9)	1011 (44.7)
Location		
Rectum (%)	1492 (27.0)	666 (29.4)
Left colon (%)	2585 (46.7)	965 (42.6)
Right colon (%)	1451 (26.2)	632 (28.0)
T factor		
T1 - 2 (%)	854 (15.4)	969 (42.8)
T3 (%)	2913 (52.7)	1004 (44.4)
T4 (%)	1763 (31.9)	290 (12.8)
Lymph node metastasis		
Negative (%)	3146 (56.9)	1463 (64.6)
Positive (%)	2362 (42.7)	800 (35.4)
Tumor size [mm]		
Median (min, max)	50 (10, 280)	35 (0, 150)
Pathological Stage		
Stage I (%)	582 (10.5)	683 (30.2)
Stage II (%)	2483 (44.9)	704 (31.1)
Stage III (%)	2443 (44.2)	876 (38.7)
Surgical resection		
D2 (%)	2293 (41.5)	694 (30.7)
D3 (%)	3227 (58.4)	1569 (69.3)
Histology		
Well differentiated (%)	2776 (50.2)	1013 (44.8)
Moderately (%)	2423 (43.8)	1097 (48.5)
Poorly (%)	136 (2.5)	145 (6.4)
Others (%)	195 (3.5)	8 (0.3)
Postoperative complication	861 (15.6)	340 (15.0)
Postoperative chemotherapy	3609 (65.3)	859 (38.0)

### Part I: Development of the prediction model

**Figure 1:**
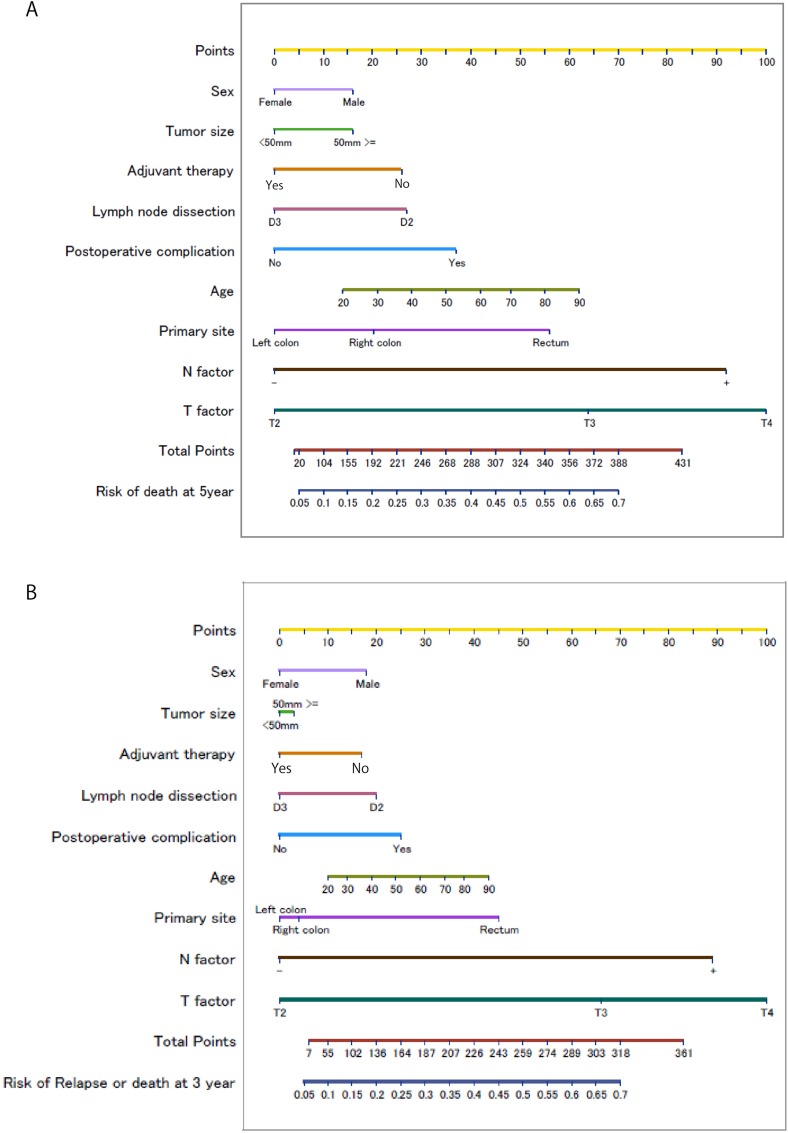
Nomograms for predicting the Overall Survival (**A**) and Disease Free Survival (**B**) Each **value** of a predictor is assigned to a point (upper scale). The total point corresponds to a probability for the 5-year death (lower scale).Each value of a predictor is assigned a score (upper scale). The total score corresponds to a probability for the 3-year recurrence or death (lower scale).

Tables 2 and 3 show the hazard ratios with Cox regression analyses after the variable selection in OS and DFS, respectively. Gender, tumor location, size, tumor depth (pathological T factor), lymph node metastasis, lymph nodes dissection, the incidence of postoperative complications and adjuvant chemotherapy were significantly associated with the OS, and all factors, except for the tumor size, were also significantly associated with the DFS. The C-index for model assessment was 0.72 (95% confidence interval [CI] 0.66–0.77) in the OS and 0.74 (95% CI 0.69–0.78) in the DFS. Figure 1 shows two nomograms predicting the OS and DFS from the results based on the selected variables with hazard ratios.

**Table 2:** Univariate and multivariable results of Cox regression analysis for overall survival

	Univariate hazard ratio (95% CI)	Multivariable hazard ratio (95% CI)	*P* value
Age [per 10 year old]	1.05 (0.98, 1.12)	1.06 (0.99, 1.14)	0.083
Sex			
Male	1.23 (1.09, 1.39)	1.15 (1.01, 1.31)	0.038
Female	1.00		
Location			
Rectum	1.37 (1.17, 1.61)	1.36 (1.14, 1.61)	0.001
Left colon	0.81 (0.70, 0.95)	0.84 (0.72, 0.99)	0.032
Right colon	1.00		
T factor			
T4	2.70 (2.15, 3.39)	2.36 (1.86, 2.99)	< 0.001
T3	1.71 (1.36, 2.14)	1.72 (1.36, 2.18)	< 0.001
T1/2	1.00		
Lymph node metastasis			
Positive	2.01 (1.77, 2.28)	2.19 (1.93, 2.49)	< 0.001
Negative	1.00		
Tumor size			
50 mm ≥	1.26 (1.11, 1.43)	1.15 (1.01, 1.31)	0.041
Less than 50 mm	1.00		
Pathological Stage			
Stage III	3.17 (2.38, 4.22)		
Stage II	1.71 (1.28, 2.30)		
Stage I	1.00		
Surgical resection			
D2	1.22 (1.08, 1.38)	1.26 (1.11, 1.42)	< 0.001
D3	1.00		
Histology			
Poorly / Others	1.37 (1.08, 1.75)		
Moderately	1.10 (0.97, 1.25)		
Highly	1.00		
Postoperative complication	1.49 (1.28, 1.73)	1.37 (1.17, 1.61)	< 0.001
Postoperative chemotherapy	0.85 (1.75, 0.96)	0.80 (0.70, 0.91)	0.001
**Model assessment in the derivation cohort**			
Number		5501	
Number of events		1018	
C-index		0.72 (0.66, 0.77)	

^*^ S_0_ (5) = 0.93778 + 0.020 = 0.95778 (5-year baseline overall survival). Baseline overall survival was updated based on the calibration plot. 95% CI was constructed based on the Wald test.

**Table 3:** Univariate and multivariable results of Cox regression analysis for disease free survival

	Univariate hazard ratio (95% CI)	Multivariable hazard ratio (95% CI)	*P* value
Age [per 10 year old]	1.04 (0.98, 1.10)	1.05 (0.99, 1.11)	0.130
Sex			
Male	1.24 (1.11, 1.38)	1.18 (1.05, 1.31)	0.004
Female	1.00		
Location			
Rectum	1.42 (1.24, 1.64)	1.46 (1.26, 1.70)	< 0.001
Left colon	0.94 (0.82, 1.07)	0.96 (0.84, 1.10)	0.564
Right colon	1.00		
T factor			
T4	2.75 (2.26, 3.35)	2.52 (2.06, 3.09)	< 0.001
T3	1.79 (1.47, 2.17)	1.84 (1.50, 2.25)	< 0.001
T1/2	1.00		
Lymph node metastasis			
Positive	2.15 (1.93, 2.89)	2.28 (2.04, 2.54)	< 0.001
Negative	1.00		
Tumor size			
50 mm ≥	1.12 (1.01, 1.25)	1.03 (0.92, 1.15)	0.591
Less than 50 mm	1.00		
Pathological Stage			
Stage III	3.28 (2.57, 4.18)		
Stage II	1.67 (1.30, 2.14)		
Stage I	1.00		
Surgical resection			
D2	1.18 (1.06, 1.31)	1.20 (1.08, 1.34)	0.001
D3	1.00		
Histology			
Poorly / Others	1.26 (1.01, 1.57)		
Moderately	1.15 (1.03, 1.28)		
Highly	1.00		
Postoperative complication	1.35 (1.18, 1.55)	1.26 (1.10, 1.45)	0.001
Postoperative chemotherapy	0.90 (0.81, 1.01)	0.85 (0.76, 0.95)	0.005
**Model assessment in the derivation cohort**			
Number		5501	
Number of events		1378	
C-index		0.74 (0.69, 0.78)	

^*^ S_0_ (3) = 0.93859 (3-year baseline disease-free survival). 95% CI was constructed based on the Wald test.

### Part II: Validation study

**Figure 2:**
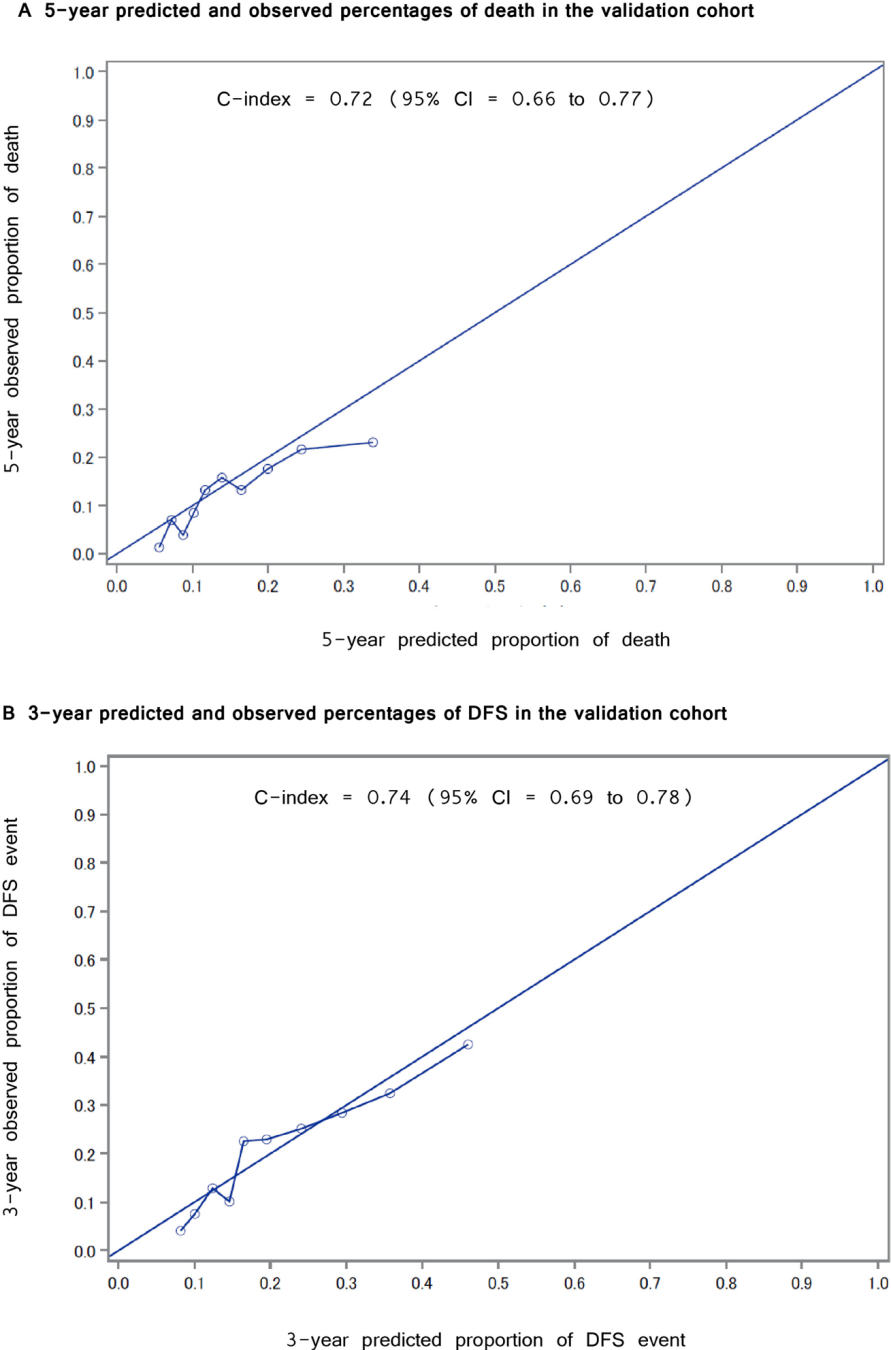
Calibration plot for Overall Survival and Disease Free Survival (**A**) The relationship of the 5-year predicted and observed percentages of death in the validation cohort (**B**) The relationship between the 3-year predicted and observed percentages of recurrence in the validation cohort.

**Figure 3:**
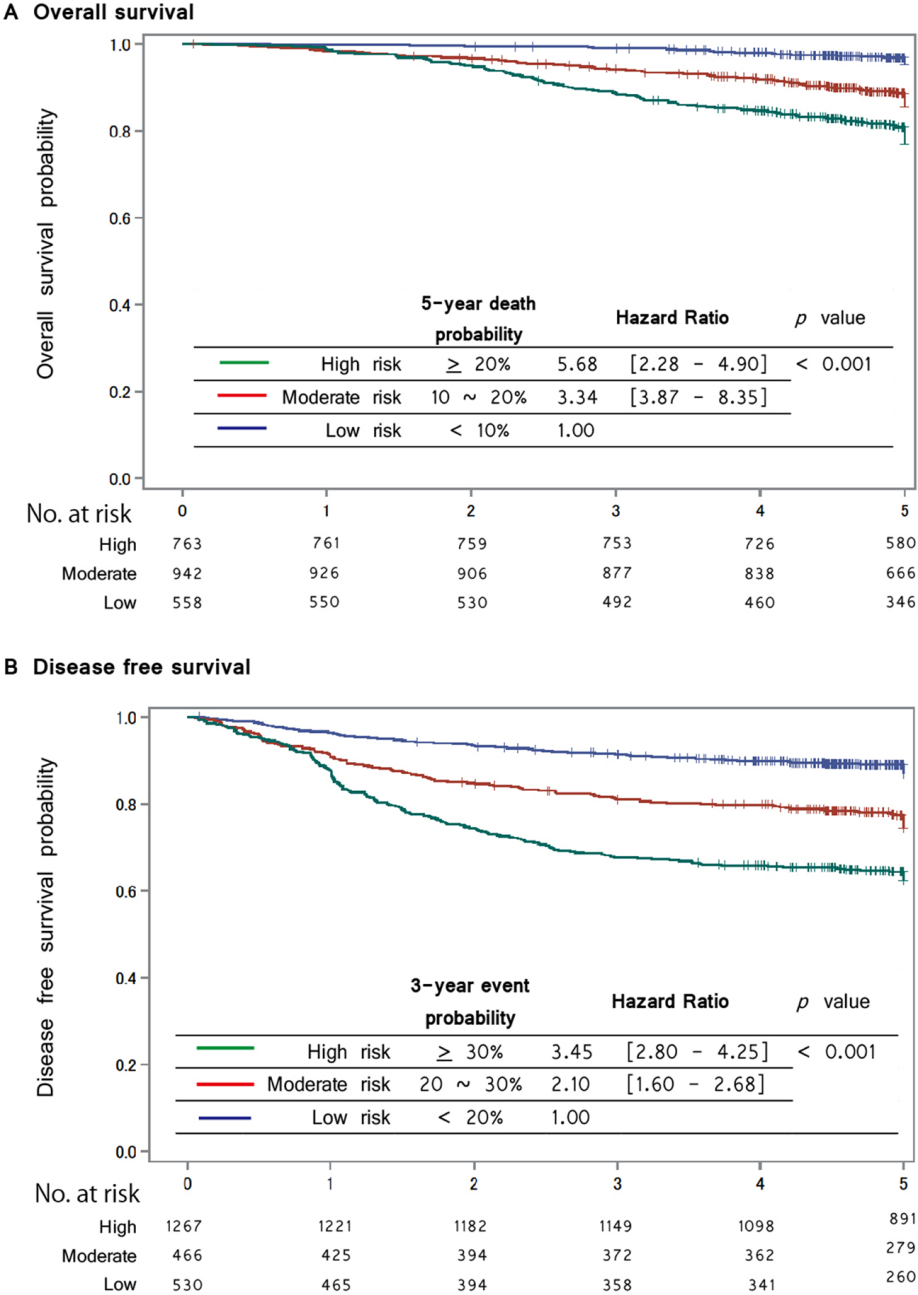
Kaplan-Meier curve stratified by risk group for the Overall Survival and Disease Free Survival in the validation cohort Kaplan-Meier curves of risk group stratification for overall survival in the validation cohort (**A**) The blue line represents a low risk; a predicted 5-year death probability of < 10%, the red line represents a moderate risk; a predicted 5-year death probability of from 10 to < 20% and the green line represents a high risk; a predicted 5-year death probability of 20% ≥. All curves were statistically different (log-rank test, *p* < 0.01). (**B**) Kaplan-Meier curves of the risk group stratification for disease free survival in the validation cohort. The blue line represents a low risk; a predicted 3-year recurrence probability of < 20%, the red line represents a moderate risk; a predicted 3-year recurrence probability of from 20 to < 30% and the green line represents a high risk; a predicted 3-year recurrence probability 30% ≥. All curves were statistically different (log-rank test, *p* < 0.01).

Figure 2 shows the calibration plot of the prediction model in the validation cohort. The plot represents the predicted five-year proportion of events; the incidence of death or recurrence is shown on the x-axis, and the actual proportion of events estimated by the Kaplan-Meier method is shown on the y-axis. To investigate the validity, the validation cohort was divided into three groups (low, moderate and high risk), thus representing the predicted 5-year death probabilities of < 10%, 10%–20% and > 20%, and 3-year recurrence or death probabilities of < 20%, 20% –30% and > 30%, respectively. Figure 3 illustrates the Kaplan-Meier curve stratified by each of the three risk group for the OS and DFS in the validation cohort. Log-rank tests to determine whether or not the prediction of events using the models reflect the OS and DFS in the validation cohort were significant for both the OS and DFS (*p* = 0.001).

## DISCUSSION

The prognostic nomogram established in this study has three strong points as follows: First, to develop the prediction model, we analyzed the pooled individual data of the long-term outcomes of the three largest and most well-followed-up phase III clinical trials. Second, the OS and DFS, which were the most important outcomes for both patients and physicians, could be predicted using several common variables such as the clinico-pathological findings or treatment information. Finally, to confirm the external validity, we identified a data set of consecutive patients from a specialized hospital. The nomogram was assumed to have high clinical exploitability.

Several previous studies developed a nomogram for patients with colon and/or rectal cancer [9]. Various target outcomes predicted by the nomogram have been reported, including postoperative complications [10, 11], operative mortality [12], distant metastasis [6, 8], peritoneal recurrence [13] and side effects of chemotherapy [14]. However, the quality of such oncological prognostic prediction models was insufficient. Indeed, previous studies are aossiciated with several critical problems, such as small sample sizes, an insufficient c-index, limited target outcomes, such as peritoneal recurrence or a lack of any validation or calibration [9]. The main reason for these shortcomings might be the difficulty in obtaining accurate clinical datasets including long-term outcomes, such as the five-year OS or relapse-free survival (RFS).

Among these previous studies, Valentini et al. [6] developed a notable prediction model and performed a validation study using large-scale individual data (*n* = 2795) that was merged from 5 major European clinical trials. One of these trials was used as a validation set and the c-index showed a relatively high (0.68 to 0.73).

Although this nomogram was acceptable for our practice, the actual application was limited to rectal cancer patients who underwent radiotherapy or chemo-radiotherapy, which is uncommon in East-Asian countries.

In the present study, the primary goal was to improve the accuracy of a prediction model for both the OS and DFS which are the most relevant and principle outcomes for patients who undergo curative resection for CRC. We enrolled 7793 patients, making this the largest-scale study ever published for predicting the OS and DFS, using individual patient data from 3 large Japanese phase III clinical trials for the training cohort and using consecutive cases in a cancer-specialized hospital for the validation cohort, and thereby established a prognostic nomogram for CRC. Furthermore, our dataset included many patients who received surgery alone or surgery plus adjuvant chemotherapy without radiotherapy. Therefore, our model could evaluate the efficacy of adjuvant chemotherapy compared with surgery alone.

We had two concerns when we conducted this study. One was that the newly developed nomogram might be associated with some selection bias, because all subjects in the derivation cohort had been enrolled according to the rigorous inclusion criteria prescribed by the protocol of clinical trials. The other was the wide range treatment period; some patients were treated over 20 years ago in this derivation cohort.

In order to reinforce these concerns, apart from the derivation, we conducted an external validation study to evaluate the generalizability of our nomogram using the dataset of consecutive patients who were treated recently in the cancer-specialized hospital. As a result, the nomogram demonstrated good discernment for the OS and DFS in risk group stratification of the validation cohort. As a result, our prediction model is therefore considered to have good external validity.

We focused on the usage of this normogram in common surgical practice, selecting only variables that physicians could easily obtain in many community hospitals in this study. To further improve the prediction ability of this normogram, it might be necessary to add molecular biological variables which are known to be prognostic predictors into the model, such as the immunohistological findings or the genetic expression of tumor specimens. Indeed, several investigators found that certain micro RNAs were useful for increasing the accuracy of a prognostic nomogram of CRC [15, 16].

Another limitation associated with this study is that our dataset might include some treatment heterogeneity, although all patients strictly fulfilled the criteria including surgery and received adjuvant chemotherapy defined by each protocol. It was impossible to adjust and control the details of the surgical procedures or chemotherapy regimens as variables in developing this model. In addition, chemoradiotherapy was not evaluated as a variable because the dataset included few cases who underwent neoadjuvant chemoradiotherapy, which is a common strategy for treating rectal cancer in Western countries, but not in Eastern ones. However, recent advances in adjuvant treatment have helped to improve the prognosis, therefore we are now planning to add other variables from recent clinical trials after refining these trial data in the future.

In conclusion, we established a clinically reliable nomogram that was able to predict the OS and DFS in individuals with CRC. The statistical models were developed based on the largest-scale dataset of phase III clinical trials ever published among all previous studies, and the external validation was confirmed using an actual practical dataset. This nomogram may become widely accepted in in surgical practice and are considered to be useful for planning follow-up examinations for individual patients after radical resection for CRC.

## MATERIALS AND METHODS

The present study was conducted in two parts. In part I, a nomogram was developed and evaluated for its internal validity. In part II, the external validity of the nomogram was investigated. To evaluate both the internal and external validities, we prepared two independent datasets for this study, as described below. This study was approved by the Institutional Review Board of the Cancer Institute Hospital and the Japanese Foundation for Multidisciplinary Treatment of Cancer.

### Part I

A total of 5,530 individual patients’ data were pooled as the development data from three open label, multicenter, randomized, phase III trials of the Japanese Foundation for Multidisciplinary Treatment of Cancer (JFMC) studies (JFMC7, JFMC15, and JFMC33). The results of each clinical trial have already been reported elsewhere [17–19]. Briefly, the JFMC 7 and JFMC 15 trials were performed to evaluate the long-term utilization of oral fluorinated pyrimidines as adjuvant chemotherapy for patients with CRC, comparing surgery alone with surgery plus postoperative chemotherapy. The main regimen of adjuvant chemotherapy was the 1-year administration of oral 5-FUs (JFMC 7–1: 200 mg/day 5-FU; JFMC 7–2 and JFMC 15: 300 mg/day 1-hexycarbamoyl-5-fluorouracil). JFMC 33 evaluated the survival benefit of receiving tegafur (UFT, 300 mg/m^2^/day as tegafur)/leucovorin (LV, 75 mg/day) for 5 consecutive days per week for 18 months compared with the standard tegafur regimen. No patients received either preoperative treatment or perioperative radiation therapy.

### Data collection and variables

To develop the prediction model, all clinically important information was extracted from the case-report forms of the targeted clinical trial or from the hospital medical records. Specifically, patients’ age, gender, primary site, tumor size, TNM stage and margin size on the resected specimen, surgical procedure, degree of lymph node dissection, residual tumor, histological findings, postoperative complications and adjuvant chemotherapy were extracted. The primary site of colon cancer was classified as the right side if the tumor was located in the cecum, ascending, hepatic flexure or transverse colon, and as the left side if the tumor was within the splenic flexure, descending, sigmoid colon or recto sigmoid junction. The survival time, timing of recurrence and site were investigated as the outcomes. OS was defined as the period between surgery and any cause of death. Disease free survival (DFS) was defined as the period between surgery and the occurrence of recurrence, 2^nd^ cancer or death, whichever came first. The data for patients who had not experienced any events were censored as of the date of the final observation.

### Statistical analyses and model development

The prediction models for the OS and DFS were developed using a Cox regression model. The data for patients who had not experienced any events were censored as of the date of the final observation. A backwards selection with *p* value of less than 0.05 was adopted to select the variables for each prediction model. The main effects and 1^st^ order interaction terms for each possible variable were considered candidates for the selection. The backward selection was repeated using 1000 bootstrap samples to adjust the final model for overfitting and exploring the reproducibility of a model [20]. Candidate variables were ranked according to their frequency of selection in the bootstrap samples. If variables were selected in > 60% of bootstrap samples, we included them as the final set of predictors in the model. We did not conduct formal sample size calculations in order to maximize the power and generalizability of the results by using all available data. Some researchers have suggested that there should be at least 10 events per candidate variable for the derivation of a model and at least 100 events for validation studies [21, 22]. Our sample size and the number of events far exceeds all approaches for determining the sample sizes and therefore this sample size is expected to provide sufficiently accurate estimates.

### Part II

A total of 2263 individual patients’ data were obtained for the external validation of the established nomogram from the Cancer Institute Hospital of Japanese Foundation Cancer Research. These were consecutive patients with histologically confirmed colorectal adenocarcinoma who had been diagnosed as having clinical stage I to III disease and who underwent radical resection from January 2005 through December 2011. The exclusion criteria were carcinoma in the appendix and the presence of another primary malignancy. Patients who underwent perioperative chemoradiotherapy were also excluded.

### Predictive performance and external validation

The predictive performance of each prediction model was evaluated based on the discrimination and calibration measurements. For the discrimination measurements, we used c-statistic [23] proposed by Pencina and D’agostino for survival model. The Kaplan-Meier curves were also depicted by three risk groups according to the estimated risk score (low, medium, and high risk). The calibration plot and its slope were also studied. As an external validation, the constructed prediction model was applied to the clinical data of the Cancer Institute Hospital and the Japanese Foundation for Multidisciplinary Treatment of Cancer. The same predictive performance was evaluated in the external validation dataset. We followed the TRIPOD guidelines [24] for developing and reporting the prediction model.

## Abbreviations

CRC: colorectal cancer
OS: overall survival
DFS: disease free survival
CI: confidence interval
JFMC: Japanese Foundation for Multidisciplinary Treatment of Cancer
TRIPOD: Transparent reporting of a multivariable prediction model for individual prognosis or diagnosis
